# Systematic Review and Meta-Analysis of 16 Randomized Clinical Trials of Radix Astragali and Its Prescriptions for Diabetic Retinopathy

**DOI:** 10.1155/2013/762783

**Published:** 2013-03-21

**Authors:** Lin Cheng, Gai Zhang, Yi Zhou, Xuejing Lu, Fuwen Zhang, Hejiang Ye, Junguo Duan

**Affiliations:** ^1^Institute of Clinical Pharmacology, Central South University, Changsha, Hunan 410078, China; ^2^National Diabetes Mellitus Clinical Research Base of TCM, Teaching Hospital of Chengdu University of TCM, Chengdu, Sichuan 610075, China; ^3^College of Clinical Medicine, Chengdu University of TCM, Chengdu, Sichuan 610075, China

## Abstract

*Objective*. To evaluate the efficacy and safety of radix astragali and its prescriptions for diabetic retinopathy. *Methods*. A computer-based online and manual search was conducted for randomized controlled trials addressing radix astragali and its prescriptions for diabetic retinopathy. *Results*. 16 RCTs involving 977 subjects and 1586 eyes were identified. Meta-analysis indicated that the effect of radix astragali and its prescriptions in improving visual acuity and fundus manifestations, lowering FBG, TG, plasma viscosity, and RAI, was superior to that of control group (WMD or OR 0.20, 0.27, −0.26, −0.36, −0.93, −1.27; 95% CI [0.09, 0.30], [0.17, 0.40], [−0.51, 0.00], [−0.60, −0.12], [−1.67, −0.20], [−2.35, −0.19]; *P* < 0.05, resp.). In contrary, the efficacy of radix astragali and its prescriptions was not superior to those of control group in descending HbA1C and TC with WMD 0.45, −0.96 and 95% CI [−1.00, 1.90], [−2.19, 0.27], *P* > 0.05, respectively. GRADE software suggested that the studies were of low methodological quality. *Conclusion*. Radix astragali and its prescriptions were superior to other treatments for diabetic retinopathy in terms of improving visual acuity and fundus manifestations, reducing FBG, TG, RAI, and plasma viscosity. The evaluated studies were of low methodological quality, indicating that the previous findings should be read with care.

## 1. Introduction

Diabetic retinopathy (DR) is a disease of retina as a complication of diabetes mellitus, resulting in loss of vision, macular edema, recurrent vitreous hemorrhages, tractional or secondary rhegmatogenous retinal detachment, and so forth [1–5]. It is characterized by the progressive microvascular complications, such as aneurysm, intraretinal edema, and intraocular pathologic neovascularization [6]. It is an accepted fact that diabetic retinopathy does not occur for at least 2–5 years after the onset of Type 1 (insulin-dependent) or type 2 (non-insulin-dependent) diabetes mellitus [7, 8]. Diabetic retinopathy is not only one of the main microvascular complications of diabetes, but also an important public health problem [9, 10]. Approximately 2.5 million people worldwide are clinically blind attributed to diabetic retinopathy [11]. In particular, DR remains a major threat to eyesight in the working age population [12, 13]. The available data suggest that the global number of people with DR will grow from 126.6 million in 2010 to 191.0 million by 2030, among them vision-threatening DR is estimated to increase from 37.3 million to 56.3 million [14]. As the incidence of diabetes gradually increases, there is the possibility that more individuals will suffer from eye complications, which, if not properly managed, may lead to permanent eye damage. 

Diabetic retinopathy can be managed by improved control of glucose and blood pressure [15, 16], pharmacological, laser, and surgical approaches [17, 18]. Principal pharmacological therapies include drugs that inhibit neovascularization, such as anti-VEGF derivatives (Avastin & Lucentis [19–21]), or drugs to relieve macular swelling such as steroidal anti-inflammatory drugs (triamcinolone acetonide [22]). Although the laser treatment with or without anti-VEGF drugs is considered the “Golden Standard” treatment of diabetic retinopathy, it is only suitable for high risk of proliferative DR or proliferative DR. Laser and surgical interventions can be very effective for diabetic retinopathy but, as yet, final choices when it draws near to proliferative diabetic retinopathy. Would there be some therapies that can prevent the progression of DR? With the distinctive traditional medical opinions and natural medicines mainly originated in herbs, the traditional Chinese medicine performed a good clinical practice and is showing a bright future in the therapy of diabetes mellitus and its complications [23]. In treating wasting-thirst/diabetes and its complications, Traditional Chinese Medicine (TCM) harbors a long history, and herb treatment is also various. Zhang et al. have done research by mining and reviewing the literature in the 2000-year history of wasting-thirst and suggested TCM has a profound understanding and effective management on diabetes and its complications [24]. Some scholars had statistical analysis of ancient Chinese treating formulas and found that radix astragali (Chinese medical herb, also named huang qi) was the most popular herb to treat diabetes [25, 26]. As in the ancient days, wasting-thirst/diabetes and its complications were treated together according to syndrome differentiation. This suggested that radix astragali was one of the most common used herb for DR. Up till now, there is insufficient evidence to support the efficacy of radix astragali and its prescriptions for diabetic retinopathy. Due to its extensive application, the authors insisted that it is necessary to do a systematic review to explore radix astragali and its prescriptions in treating diabetic retinopathy. 

This study was designed to assess the efficacy and safety of radix astragali and its prescriptions in treating diabetic retinopathy by conducting literature reviews in Chinese and English databases for RCTs. In this systematic review and meta-analysis, multiple publications reporting the same group of participants, or their subsets, were excluded. 

## 2. Data and Methods

### 2.1. Search Strategy

The following sources were searched up to Septemper 2010: The Cochrane Library, Medline, EMBASE, Current Controlled Trials, Chinese Biomedical Literature Database (CBM), China National Knowledge Infrastructure (CNKI), VIP database, Wanfang database, 1980−2010.06 inclusive. We also conducted manual search of relevant journals, symposia, degree papers, and conference proceedings to retrieve relevant trials for cross checking some data that may be missed on electronic devices. The reference lists of papers were checked for further relevant publications, and experts were asked for information concerning any additional trials. Personal contact was made with the authors of the studies, if necessary, to request for additional data.

Search terms used to search RCTs were (“diabetic retinopathy” OR “eyeground changes” OR “fundus disease” OR “fundus changes” OR “eye disease”) AND (“herb” OR “radix astragali” OR “astragalus mongholicus” OR “astragalus membranaceus” OR “astragalus” OR “huangqi”) AND (“randomized controlled trial” OR “controlled clinical trial” OR “random” OR “randomly” OR “randomized” OR “control”).

### 2.2. Inclusion and Exclusion Criteria

#### 2.2.1. Type of Studies

Our paper was restricted to RCTs that compare radix astragali and its prescriptions for diabetic retinopathy to a control group, including placebo, no treatment (blank), or conventional treatment (western medicine treatment), other herb treatment (except radix astragali)) but not including laser treatment and acupuncture. Observational studies, reviews, animal studies, nonrandom or quasirandom studies, studies not taken visual acuity, as one of outcome measures or the description of visual acuity was not clear enough for statistics, were excluded. No restriction was imposed on studies with respect to publication types, blinding, and the type of design such as parallel or cross over. Crossover trials were evaluated as long as outcome data were available for each treatment segment prior to the crossover.

#### 2.2.2. Type of Participants

This study evaluated retinopathy induced by type 1 and type 2 diabetic mellitus despite of gender, age, or nationality. Diabetic retinopathy could be either nonproliferative diabetic retinopathy (NPDR) or proliferative diabetic retinopathy (PDR). This study excluded participants combined with other ocular diseases like glaucoma or severe cataract. Pregnant and lactating women were also excluded.

#### 2.2.3. Type of Interventions. 

All forms of radix astragali and its prescriptions intervention regardless of produced pharmaceutical factories were considered as the treatment group. Placebo-controlled, no treatment (blank), or conventional treatment (doxium, neuroprotection, etc.), and other herb treatment (Flos Carthami, etc.) were considered as the comparison group. Laser photocoagulation, acupuncture, or both groups that used radix astragali were excluded. 

#### 2.2.4. Type of Outcome Measures

The primary outcomes were visual acuity and fundus manifestations, and secondary outcomes assessed were Fasting Blood Glucose (FBG), HbA1C, TC, TG, erythrocyte sedimentation rate (ESR), plasma viscosity, RBC aggravation index (RAI), and adverse events. 

### 2.3. Data Extraction and Quality Assessment

Data extraction and quality assessment were independently fulfilled by two authors. For each study an author was nominated at random as data extractor, checker, or adjudicator and no one should be data extractor on a paper they authored. Where there was uncertainty regarding eligibility between authors, it was resolved by discussion and consensus or the third party, if necessary. Personal contact was made with authors of published studies when papers contained insufficient information to make a decision about eligibility. Besides looking through abstracts and full-text paper, the authors also paid attention to reference to cull irrelevant citations. The data extraction and quality assessment of all studies were done by two authors following the detailed descriptions of these categories as described in *Assessing Risk of Bias in the Cochrane Handbook for Systematic Reviews of Interventions by Higgins and Altman* [27]. The methodological quality of trials was assessed by the GRADE profiler 3.2.2 software which moved from the evidence to a accommodation for systematic reviews [28] and guidelines in chapter 8 of the Cochrane Handbook for Systematic Reviews of Interventions. And the clinical data were analyzed by RevMan Manager (version 5.0.16 Cochrane Collaboration, Oxford) with the random effect analysis mode. Data analysis followed the guidelines in chapter 9 of interventions. A structured pro forma was used for data extraction (Table 1). According to the type of outcome indexes, measurement data were assessed by weighted mean difference (WMD) and 95% confidence interval (95% CI). Before combining data, heterogeneity was estimated by Chi-square test and *I* ^2^ test. 

Some studies used decimal point method, that is, Snellen fraction, to value the changes of visual acuity, while the others used five-score method. The authors divided the visual acuity evaluation into two subgroups. One was five-score group; the other was Snellen fraction group. Mean changes from baseline were calculated by posttreatment mean minus pretreatment mean, while SD(standard deviation) values were estimated by the following formula:   |A^2-B^2|  +AB (*A*: data pretreatment; *B*: data posttreatment). The final data would round to two decimal places with a decimal place of  0.4 or less got rounded down, while one of  0.5 or more got rounded up.

## 3. Results

### 3.1. Study Description

**Figure 1:**
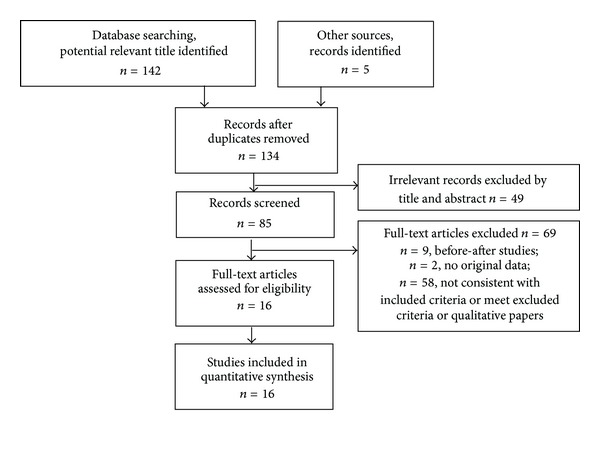
Flowchart showing the number of studies evaluated and excluded from the systematic review.

A total of 147 studies were initially identified, 142 of them came from electronic database and 5 of them came from other sources. With full-text review, only 16 studies, involving 977 subjects and 1586 eyes (832 eyes in experimental group and 754 eyes in control group) were in accordance with our inclusion criteria and not meet the exclusion criteria. Among the 16 studies, four [30, 36, 37, 44] came from master degree paper database, the rest were journal papers.  Figure 1 summarized the search results in flowchart. All trials were conducted in China and published in Chinese. 

### 3.2. Methodological Quality

The quality assessments were summarized in Table 2. Three studies described adequate methods of randomization using a computer-generated randomization [31] or random number tables [30, 36, 44]. The others did not describe the sequence generation process. Except for one study [31], all evaluated trials received allocation scores of “unclear” as they did not have clear descriptions of their method of allocation concealment. Three studies [30, 31, 36] mentioned blinding; the rest studies were not blinded. One study [31] mentioned loss of participants due to loss of followup. The others had no description of participant dropout. All trials provided patient characteristics in the study group, but lack of data to determine whether these studies had selective outcome reporting or other source of bias. GRADE profiler rated the quality of the evidence low, which indicated that further investigations might influence the confident intervals of this meta-analysis and the result would likely be reversed.

### 3.3. Characteristics of Included Studies

The characteristics of include studies were shown in Table 1. Among the sixteen studies, all used visual acuity to assess the effect of radix astragali and its prescriptions for diabetic retinopathy, only six reported the adverse events. All the experimental interventions were oral administration. The control group intervention included gliclazide, other herbs, placebo, or no treatment. A continuous numerical scale, such as visual acuity, FBG, HbA1C, TC, TG, or plasma viscosity, was used in all include studies. Studies used multiply scales to rate the degrees of visual acuity were not evaluated due to the difficulty to unify standard. Total effects and fundus changes were not pooled because different studies use different standards. So other indexes such as hematocrit (HCT), whole blood viscosity, were not pooled due to the small number of studies and the clinical heterogeneity of these trials. All the participants came from inpatient and/or outpatient department of ophthalmology or endocrinology. The age of participants varied from 43 to 75.75 years. The course of treatment ranged from four weeks to ten months. The experimental interventions comprised huangqi decoctions, pills, and capsules. The control interventions included placebo, various western drugs (e.g.: gliclazide pills), other herbs, and no DR treatment (DM conventional treatment). 

### 3.4. Outcome Measurements

#### 3.4.1. Visual Acuity

**Figure 2:**
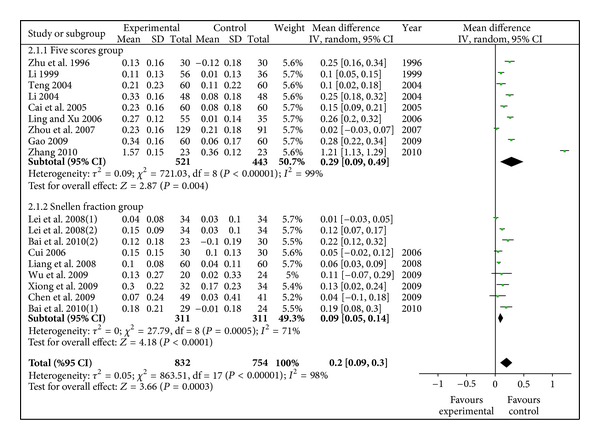
Meta-analysis of visual acuity (radix astragali group versus control group).

16 studies involving 1586 eyes with 832 in experimental group and 754 in control group were synthetized in meta-analysis for visual acuity. The total result in Figure 2 showed that the improvement of visual acuity in radix astragali group was significantly better than that of control group (WMD 0.20, 95% CI [0.09, 0.30]) with a degree of heterogeneity (*X* ^2^ = 863.51, *P* < 0.10, *I* ^2^ = 98%). In each subgroup, the incorporated data revealed that visual acuity in radix astragali group was superior to control group (WMD 0.29, 95% CI [0.09, 0.49]; (WMD 0.09, 95% CI [0.05, 0.14]) with heterogeneity (*X* ^2^ = 721.03, *P* < 0.10, *I* ^2^ = 99%; *X* ^2^ = 27.79, *P* < 0.10, *I* ^2^ = 71%).

#### 3.4.2. Fundus Manifestations

**Figure 3:**
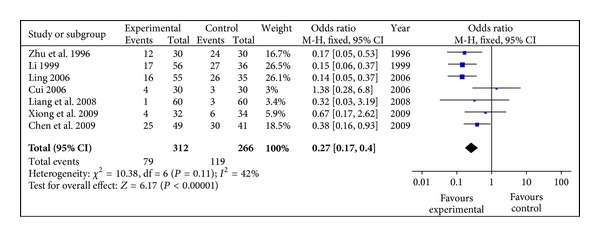
Meta-analysis of fundus manifestations (radix astragali group versus control group).

7 studies had reported fundus manifestations (Fundus Fluorescein Angiography) involving 578 eyes with 312 in experimental group and 266 in control group. Inefficiency rate was synthetized in the meta-analysis to test effect on fundus manifestations. The result in Figure 3 showed that difference of fundus improvement between radix astragali group and control group was significantly (OR 0.27, 95% CI [0.17, 0.40], *P* < 0.01) with homogeneity (*X* ^2^ = 10.38, *P* > 0.1, *I* ^2^ = 42%). 

#### 3.4.3. FBG (mmol/L)

**Figure 4:**
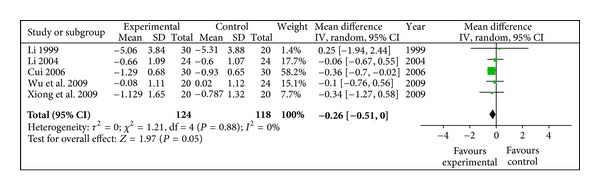
Meta-analysis of FBG (radix astragali group versus control group).

Only four evaluated studies involving 242 participants reported the reduction of FBG. Meta-analysis shown in Figure 4 suggested that the efficacy of radix astragali and its prescription was superior to the control group (WMD −0.26, 95% CI [−0.51, −0.00]), with no heterogeneity (*X* ^2^ = 1.21, *P* > 0.10, *I* ^2^ = 0%).

#### 3.4.4. HbA1C (%)

**Figure 5:**
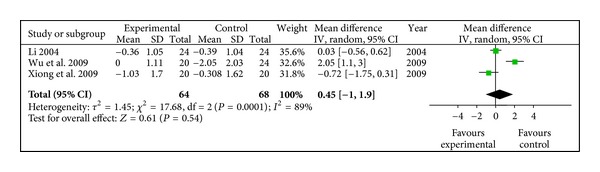
Meta-analysis of HbA1C (radix astragali group versus control group).

Three studies indicated that radix astragali group was not better than the control group (WMD 0.45, 95% CI [−1.00, 1.90]) in lower down HbA1C, with a degree of heterogeneity (*X* ^2^ = 17.68, *P* < 0.10, *I* ^2^ = 89%), shown in Figure 5.

#### 3.4.5. TC (mmol/L)

**Figure 6:**
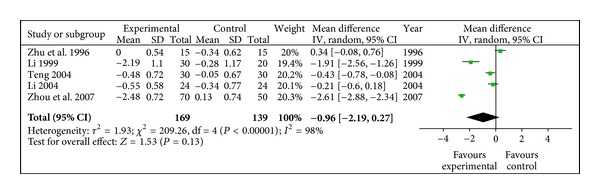
Meta-analysis of TC (radix astragali group versus control group).

Reduction of Total Cholesterol in radix astragali group was not significantly better than that of control group (WMD-0.96, 95% CI [−2.19, 0.27]) with a degree of heterogeneity (*X* ^2^ = 209.26, *P* < 0.10, *I* ^2^ = 98%), shown in Figure 6.

#### 3.4.6. TG (mmol/L)

**Figure 7:**
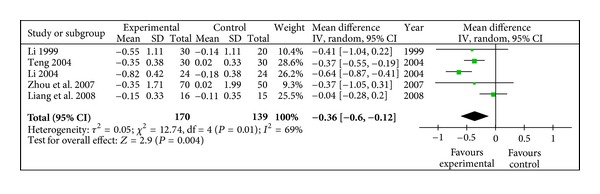
Meta-analysis of TG (radix astragali group versus control group).

Decrease of Triglyceride in radix astragali group was significantly better than that of control group (WMD −0.36, 95% CI [−0.60, −0.12]) with heterogeneity (*X* ^2^ = 12.74, *P* < 0.10, *I* ^2^ = 69%), shown in Figure 7.

#### 3.4.7. Plasma Viscosity** **(mPa/s)

**Figure 8:**
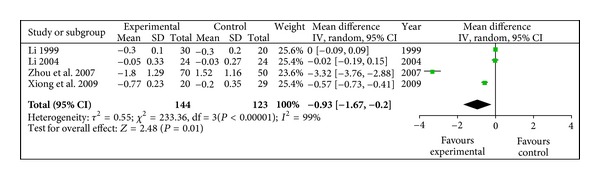
Meta-analysis of plasma viscosity (radix astragali group versus control group).

Decrease of plasma viscosity in radix astragali group was significantly better than that of control group (WMD −0.93, 95% CI [−1.67, −0.20]) with heterogeneity (*X* ^2^ = 233.36, *P* < 0.10, *I* ^2^ = 99%), shown in Figure 8.

#### 3.4.8. RAI

**Figure 9:**
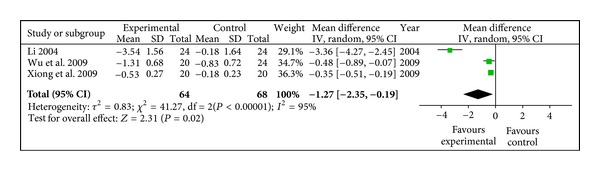
Meta-analysis of RAI (radix astragali group versus control group).

RAI in radix astragali group was significantly descending compared with control group (WMD −1.27, 95% CI [−2.35, −0.19]) with heterogeneity (*X* ^2^ = 41.27, *P* < 0.10, *I* ^2^ = 95%), shown in Figure 9.

### 3.5. Publication Bias

**Figure 10:**
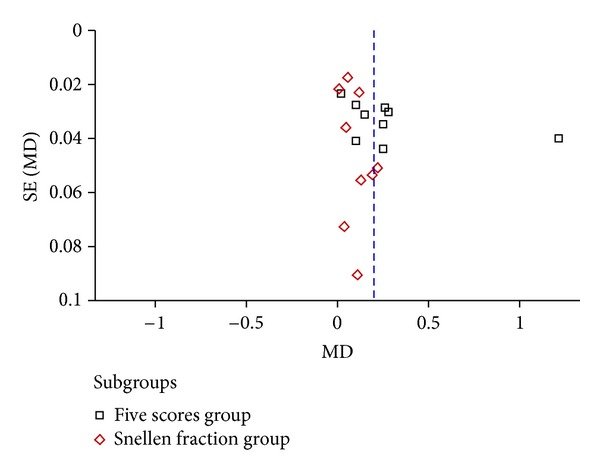
Funnel plot of publication bias.

Publication bias was assessed using the Begg's rank correlation method. Results are presented as a funnel plot (Figure 10). The funnel plot appeared to be asymmetric. All of the evaluated studies lay within the 95% CI and were uniformly distributed around the horizontal line. Since all the evaluated studies were conducted in China and published in Chinese, language bias and geographical bias may result in publication bias.

### 3.6. Sensitivity Analysis

**Figure 11:**
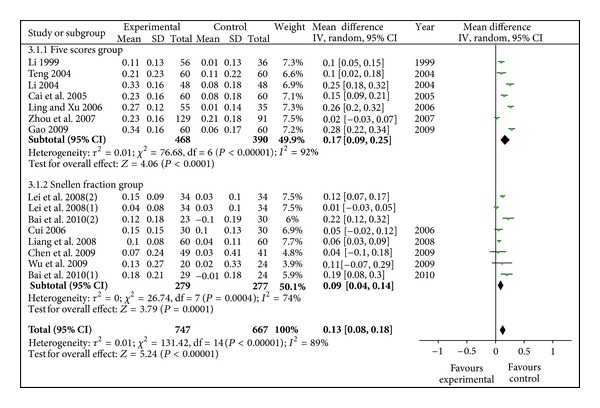
Sensitivity analysis of visual acuity (radix astragali group versus control group, 13 studies evaluated).

In the primary analysis, random-effect models were applied for the analysis of outcome measures of visual acuity, FBG, HbA1C, TC, TG, plasma viscosity and RAI. Only visual acuity had enough studies for making sensitivity. We recalculated the sensitivity analysis (see Figure 11) after removing some low-quality studies [40, 42] for quasi-random method; [41] for its far deviation) and the application of fixed-effect models; it were found that the overall estimates were virtually identical and the 95% CI were similar between the sensitivity analysis (visual acuity: WMD 0.13, 95%CI [0.08, 0.18]) and the primary analysis (visual acuity: WMD 0.20, 95%CI [0.09, 0.30]).

### 3.7. Adverse Events

We are also interested in investigating any possible adverse events in such studies. Only three papers [29, 31, 37] reported adverse reactions, but no adverse events had been observed in both groups.

## 4. Discussion

Diabetic retinopathy has a serious impact on patients in terms of visual impairment, and disturbance of glucose, lipid, and poor quality of life. In many countries, conventional western medicine therapy is considered as the standard treatment for diabetic retinopathy. Most patients are usually treated by improved glycemic control and blood pressure control [16, 45, 46], calcium dobesilate, anti-VEGF therapy, laser photocoagulation, or vitreoretinal surgeries. However, these managements are far from clinical satisfaction. Taking anti-VEGF therapy for example, it is limited by short-lived effects and needs repeated injection and reinforced treatment [47–49]. Thereby, it is essential to introduce another effective and safe unconventional treatment of diabetic retinopathy for future drug development. The previous researches revealed that radix astragali was one of the most common used herb for DR. Hence, we have conducted this systematic review to compare the efficacy of radix astragali and its prescriptions with that of conventional western drugs, placebo, or other herbs in the management of DR.

### 4.1. Result Analysis

In this systematic review, a number of English and Chinese literature databases were searched. We applied a comprehensive search strategy with a language of English and Chinese. Finally, 16 studies involving 977 participants and 1586 eye (832 eyes in experimental group and 754 eyes in control group) were finally identified. The authors incorporated visual acuity, fundus manifestations, FBG, HbA1C, TC, TG, plasma viscosity, and RAI as the outcome measures to perform meta-analysis. The results revealed that, with regard to visual acuity, fundus manifestations, FBG, TG, plasma viscosity, and RAI, the experimental group (radix astragali and its prescriptions) was superior to the control group (placebo, blank, other herbs, and conventional western drugs). While in the perspective of HbA1C and TC, no evidence had been found to support that the experimental group was better than the control group. Moreover, sensitivity analysis of visual acuity of all evaluated studies supported radix astragali and its prescriptions had better effect in improving eyesight. The results showed that radix astragali and its prescriptions had some potential as future therapeutic targets in DR; the mechanism might lay on reducing FBG, TG, plasma viscosity and RAI to perform the beneficial function of improving visual acuity. Although it showed no favour of reducing HbA1C and TC, it might possibly attributed to small evaluated literatures and observing time. Plus, methodological quality assessment suggested the evidence was low, so the results should be read with care.

### 4.2. Limitations of This Study

In general, the evidence of radix astragali and its prescriptions for diabetic retinopathy is positive. These data suggested that radix astragali and its prescriptions alleviated diabetic retinopathy in the perspective of improving visual acuity and fundus manifestations, reducing FBG, TG, RAI, and plasma viscosity, which seemed to be superior to control group. However, these results seem to be misread and should be carefully explained, due to following factors. (1) Design of studies: these clinical trials seldom provided details of their randomized techniques (except three studies) and clear statement of allocation concealment (except one study). In addition, only one study described loss of data, most of the studies not mentioned the follow-up length. Therefore, the significant difference between the experimental group and the control group might be a result of low quality of methodologies. (2) Blinding of studies: this study had a limitation in that it had been obvious to investigators that which group was being tested for its efficacy, which group's treatment was merely an ordinary or conventional treatment. Only one study [31] mentioned the double blinding, two studies [30, 36] mentioned the single blinding, the rest had no description on blinding. Consequently, the experimental group, having read about the purpose of the study and about the interventions, must have had heightened expectation relative to the control group. Therefore, there seemed to have relative potential bias with regard to investigators or participants. (3) Characteristics of participants: variance among age, basic vision, different type of DM, treating course in these studies were too great to have no bias in baseline. It is also hard for authors to identify diabetic macular edema (DME) in evaluated patients, because DME is very important factor that will have a dramatic effect on changes of visual acuity. If the patient has DME, the visual acuity in radix astragali group may be overestimated after treatment while the visual acuity in control group will abirritate the effect of radix astragali. The authors have thought of dividing patients into PDR and NPDR subgroups. However, the data were not reported in the evaluated studies. (4) Publication bias: the funnel plot detected publication bias. It may lie in the evaluated studies which were all conducted in China and published in Chinese. So this study was limited to national health boundaries regarding leaking out-of-area data. 

Besides, systematic reviews are secondary research (“research on research”). The object of scrutiny is not the effect of radix astragali and its prescriptions for diabetic retinopathy, but the literatures on this topic. So while this type of research cannot be used to “prove” a hypothesis, the compiled data can encourage the generation of new hypotheses that can then be tested prospectively, with new data. So, systematic reviews are best suited to hypothesis-generation. Besides, comparisons in systematic reviews should be planned, based on directional hypotheses, and limited to a reasonable number of studies. Consequently, the author strongly recommended researchers should firstly come to a consensus about the most appropriate specific and defined protocol for this type of study and use the same protocol for future studies.

On the other hand, the methodological quality of evaluated studies in this paper was generally low. To improve the quality, future researchers should report the method of randomization and allocation concealment, use binding as far as possible. To further verify the efficacy and safety as to obtain the best evidence outside, larger, multicenter clinical studies with prolonged follow-up time are urgently desired. 

## 5. Conclusion 

The combined results showed that radix astragali and its prescriptions had positive effect to improve DR patients' visual acuity and fundus manifestations and to reduce FBG, TG, plasma viscosity, and RAI. At the same time, the result also showed that current evidence could not prove astragalus preparation treatment group was better than control group in regulating HbA1C and TC. Radix astragali and its prescriptions had shown some potential as future therapeutic targets in DR; however, the evidence was not sufficient due to low quality of all included studies. Thereby well-designed, large-scale high-quality randomized controlled trials are warranted for stronger evidence. And information on adverse effects should also be provided in future trials. 

## Figures and Tables

**Table 1:** Characteristics of evaluated studies.

					Baseline of participants				Interventions		Outcomes
Trials	Simple size	Eyes	Random method	DM type	Gender	DR stage	Age (yr)	Period	Experimental group	Control group
Bai et al., 2010 (1) [29]	37 (15/12)	29/24	NMT	2	(9 M : 6 F)/(7 M : 5 F)	Moderate NPDR	55 ± 8/50 ± 9	12 W	50 mL huangqi decoction, bid	None	VA

Bai et al., 2010 (2) [29]	29 (13/16)	23/30	NMT	3	(4 M : 9 F)/(5 M : 11 F)	Severe NPDR	54 ± 11/59 ± 9	12 W	50 mL huangqi decoction, bid	None	VA

Gao, 2009 [30]	60 (30/30)	60/60	RNT	2	(15 M : 15 F)/(14 M : 16 F)	I–III	56.33 ± 8.6/54.16 ± 7.1	8 W	300 mL huangqi decoction, bid	6 g shihuyeguang pill (ingredient not including huangqi), tid	VA

Wu et al., 2009 [31]	44 (20/24)	20/24	Computer	2	(11 M : 9 F)/(8 M : 16 F)	NPDR	55.04 ± 7.05/56.07 ± 6.82	12 W	4 pills of huangqi capsule, tid + 2 pills placobo calcium dobesilate capsule, tid	4 pills placobo huangqi capsule, tid + 2 pills calcium dobesilate capsule, tid	VA, FBG, HbA1C, RAI

Ling and Xu, 2006 [32]	51 (30/21)	55/35	NMT	2	(20 M : 10 F)/(14 M : 7 F)	I–IV	57.2/56.8	12 W	Huangqi decoction, bid	2 tablets of pancreatic kinionogenase enteric-coated Tablet, tid	VA, FM

Zhou et al., 2007 [33]	120 (70/50)	129/91	NMT	NMT	(30 M : 40 F)/(21 M : 29 F)	NPDR	53.8/54.7	12 W	1.5 g huangqi capsule, tid	120 IU pancreatic kinionogenase enteric-coated tablet, tid	VA, TC, TG, PV

Cai et al., 2005 [34]	60 (30/30)	60/60	NMT	2	(18 M : 12 F)/(17 M : 13 F)	I–III	56.3 ± 11.3/60.2 ± 9.2	16 W	Huangqi decoction, bid	10 mL xueshuantong injection, ivgtt, qd	VA

Liang et al., 2008 [35]	60 (30/30)	60/60	NMT	2	(12 M : 18 F)/(14 M : 16 F)	NPDR and PDR	59.6/61.2	8 W	Huangqi decoction, bid	500 mg doxium, bid	VA, FM, TG

Teng, 2004 [36]	60 (30/30)	60/60	RNT	NMT	24 M : 36 F	NPDR	NMT	10 Mos	250 mL huangqi decoction, bid	500 mg doxium, bid	VA, TC, TG

Li, 2004 [37]	48 (24/24)	48/48	NMT	NMT	(14 M : 10 F)/(13 M : 11 F)	I–III	(58.72 ± 6.74)/(54.81 ± 6.84)	3 Mos	100 mL huangqi decoction, bid	2 tablets of ifrarel, tid	VA, FBG, HbA1C, TC, TG, PV, RAI

Chen et al., 2009 [38]	46	49/41	NMT	2	NMT	NPDR	NMT	3 Mos	Huangqi decoction, bid	500 mg calcium dobesilate capsule, bid	VA, FM

Lei et al., 2008 (1) [39]	68 (34/34)	34/34	NMT	NMT	(16 M : 18 F)/(19 M : 15 F)	I–III	56.4 ± 7.3/57.5 ± 6.6	2 Mos	Huangqi decoction, qd	50 mg aspirin, qd	VA

Lei et al., 2008 (2) [39]	68 (34/34)	34/34	NMT	NMT	(18 M : 16 F)/(19 M : 15 F)	I–III	59.6 ± 7.7/57.5 ± 6.6	2 Mos	Huangqi decoction, qd + 50 mg aspirin, qd	50 mg aspirin, qd	VA

Xiong et al., 2009 [40]	40 (20/20)	32/34	HN	2	(9 M : 11 F)/(10 M : 10 F)	I–III	45–70/43–70	90 D	Huangqi decoction, bid	500 mg doxium capsule, bid	VA, FM, FBG, HbA1C, PV, RAI

Zhang, 2010 [41]	46 (23/23)	23/23	NMT	NMT	(10 M : 13 F)/(11 M : 12 F)	NMT	52.12 ± 3.70/55.20 ± 4.11	4–8 W	6 g huangqi pills, bid	Conventional treatment of DM	VA

Zhu et al., 1996 [42]	30 (15/15)	30/30	HN	2	(8 M : 7 F)/(7 M : 8 F)	I–III	56.30 ± 11.30/58.20 ± 9.20	6 Mos	6 pills of huangqi pill, tid	Conventional treatment of DM	VA, FM, TC

Li et al., 1999 [43]	50 (30/20)	56/36	NMT	1, 2	NMT	I–V	NMT	2 Mos	20 mL huangqi decoction, tid	80 mg gliclazie tablet, bid	VA, FM, TC, TG, PV

Cui, 2006 [44]	60 (30/30)	30/30	RNT	2	(12 M : 18 F)/(13 M : 17 F)	NPDR	60.25 ± 5.40/58.70 ± 5.75	90 D	1 dosage of huangqi decoction, qd	100 mg ifrarel, tid, for 20 days, withdraw 10 days and for 3 consecutive courses	VA, FM, FBG

NMT: not mention it, VA: visual acuity, FM: fundus manifestations, PV: plasma viscosity, RNT: random number table, HN: hospitalization number, (1)(2): 2 individual trials conducted in 1 paper.

**Table 2:** Quality assessment of evaluated studies.

Items	Gao, 2009 [30]	Bai et al., 2010 [29]	Wu et al., 2009 [31]	Ling and Xu, 2006 [32]	Zhou et al., 2007 [33]	Cai et al., 2005 [34]	Liang et al., 2008 [35]	Teng, 2004 [36]	Li, 2004 [37]	Chen et al., 2009 [38]	Lei et al., 2008 [39]	Xiong et al., 2009 [40]	Zhang, 2010 [41]	Zhu et al., 1996 [42]	Li et al., 1999 [43]	Cui, 2006 [44]
Adequate sequence generation	Yes	UC	Yes	UC	UC	UC	UC	Yes	UC	UC	UC	UC	UC	UC	UC	Yes
Allocation concealment	No	No	Yes	No	No	No	No	No	No	No	No	No	No	No	No	No
Blinding method	Single blinding	No	Double blinding	No	No	No	No	Single blinding	No	No	No	No	No	No	No	No
Incomplete outcome data addressed	No	No	Yes	No	No	No	No	No	No	UC	No	No	No	No	No	No
Selective outcome reporting	UC	UC	UC	UC	UC	UC	UC	UC	UC	UC	UC	UC	UC	UC	UC	UC
Other source of bias	UC	UC	UC	UC	UC	UC	UC	UC	UC	UC	UC	UC	UC	UC	UC	UC

UC: unclear.
